# A Review on the Relationship between Aspirin and Bone Health

**DOI:** 10.1155/2017/3710959

**Published:** 2017-01-09

**Authors:** Kok-Yong Chin

**Affiliations:** Department of Pharmacology, Universiti Kebangsaan Malaysia Medical Centre, Cheras, Kuala Lumpur, Malaysia

## Abstract

Aspirin is a cyclooxygenase inhibitor commonly used in primary prevention of cardiovascular diseases and cancers. Its users are elderly population susceptible to osteoporosis. It also inhibits the synthesis of prostaglandin E_2_ essential in bone remodeling. This prompts the question whether it can influence bone health among users. This review aimed to summarize the current literature on the use of aspirin on bone health. A literature search on experimental and clinical evidence on the effects of aspirin on bone health was performed using major scientific databases. In vitro studies showed that aspirin could enhance the survival of bone marrow mesenchymal stem cells, the progenitors of osteoblasts, and stimulate the differentiation of preosteoblasts. Aspirin also inhibited the nuclear factor kappa-B (NF*κ*B) pathway and decreased the expression of receptor activator of NF*κ*B ligand, thus suppressing the formation of osteoclast. Aspirin could prevent bone loss in animal models of osteoporosis. Despite a positive effect on bone mineral density, the limited human epidemiological studies revealed that aspirin could not reduce fracture risk. A study even suggested that the use of aspirin increased fracture risk. As a conclusion, aspirin may increase bone mineral density but its effect on fracture prevention is inconclusive. More data are needed to determine the effects of aspirin and bone health in human.

## 1. Introduction

Homeostasis of the skeletal tissue is controlled by three types of bone cells from different lineages. Osteoblasts responsible for bone formation are derived from mesenchymal stem cells expressing transcription factors runt-related factor 2 and osterix. Osteoclasts in command of bone resorption are derived from haematopoietic stem cells and they expressed unique markers such as calcitonin receptor (CTR), tartrate-resistant acid phosphatase (TRAP), and cathepsin-K (CTSK). Osteocytes are terminally differentiated osteoblasts sepulchered in the bone matrix and they are capable of both building and digesting the bone and influencing the activities of other bone cells. The crosstalk between osteoblasts and osteocytes is mediated by various intercellular signalling molecules. Osteoblasts secrete receptor activator of nuclear factor kappa-B (RANK) ligand (RANKL), which binds with RANK on preosteoclasts and stimulates their differentiation into osteoclasts. Osteoblasts also release osteoprotegerin (OPG) acting as a decoy receptor to bind with RANKL, thus inhibiting the differentiation of osteoclasts (reviewed in [1, 2]).

Bone metabolism is influenced by many endogenous and exogenous factors [3]. Prostaglandin E_2_ (PGE_2_), the precursors for inflammatory cytokines synthesized by cyclooxygenase (COX), is one of the factors affecting bone metabolism [4]. It is essential for the formation of osteoblast and bone tissue and influences the formation of osteoclast, possibly by altering the RANKL-OPG axis [5–9]. Prostaglandin E_2_ is also vital in the transduction of mechanical signals in osteocytes [10].

Aspirin is the prototype drug for nonsteroidal anti-inflammatory drugs (NSAIDs), with known antipyretic, analgesic, and inflammatory effects [11, 12]. It inhibits all isoforms of COX by forming irreversible covalent bond with the hydroxyl group for serine 530 (acetylation), thus blocking the access of arachidonic acid to the enzymes [11, 12]. Due to its adverse side-effect of gastrointestinal bleeding, aspirin is replaced by selective COX-2 inhibitors for the treatment of fever, pain, and inflammation [13]. Nowadays, it is more commonly used at low doses to prevent cardiovascular events in high-risk individuals due to its antiplatelet effects [14, 15]. Some studies also suggested that low-dose aspirin could reduce the risk for colorectal cancer [15]. Data from the National Health Interview Survey (United States) in 2010 indicated that, among 27,157 subjects aged 18 years and above, 19% were regular users of aspirin (at least three times a week for more than three months) [8]. The number of regular users had increased by 57% compared to 2005, probably due to its widely reported protective effects on cardiovascular system [8]. In view of the prevalent use of aspirin among elderly population susceptible to bone loss and its effects on COX that produces PGE_2_, a regulator of bone metabolism, the question on whether aspirin could affect bone health arises.

This review aimed to summarize the current evidence on the effects of aspirin on the skeletal system. Osteoporosis is a metabolic skeletal disease affecting the elderly, characterized by an imbalanced bone remodeling, whereby the rate of bone resorption is greater than bone formation [16]. It leads to osteoporotic fracture, which causes significant mortality and morbidity among the patients [17–19]. Since the elderly population is likely to use aspirin for primary prevention of diseases, it is important to know the influence exerted by aspirin on their bone health.

## 2. Literature Search

Literature search was performed within the period 15/7/2016–15/8/2016 with PubMed, Scopus, and Web of Science using keywords “aspirin OR salicylate acid” AND “osteoporosis”. Relevant original research articles written in English or Mandarin were retrieved. Studies on human, animal, and cellular models were included in this review.

## 3. The Effects of Aspirin on Bone Cells

Aspirin dose-dependently reduced the formation of TRAP positive cells from RAW 264.7 macrophage cell line and the mRNA expression of osteoclast markers, namely, TRAP, CTSK, MMP-9, and CTR [20]. The inhibitory effects of aspirin on osteoclast-like cells were exerted thought the nuclear factor kappa-B (NF*κ*B) system [20]. The NF*κ*B is a transcription factor important in the synthesis of inflammatory cytokines [21]. Its activation requires the degradation of its natural inhibitor, IKB*α*, and its translocation into the nucleus to activate transcription of its target genes [22]. Aspirin suppressed phosphorylation and degradation of IKB*α* and phosphorylation of p50/p65 and the related cell signalling molecules ERK, p38, and JNK [20]. The nuclear translocation of p65 was also inhibited by the incubation of RAW 264.7 cells with aspirin [20].

Aspirin could promote the survival of bone marrow mesenchymal stem cells (BMMSC), the progenitor of osteoblasts [23]. Activated T-lymphocytes induced the apoptosis of BMMSC by Fas/Fas ligand (FasL) interaction, whereby T cells expressed FasL and osteoblasts expressed Fas [23]. Aspirin was found to prevent Fas-induced apoptosis of BMMSC [23]. It also enhanced the activity of telomerase and increased the telomere length of BMMSC, thus promoting their survival [23]. At the same time, it augmented the expression of RUNX2, ALP, and osteocalcin and facilitated the degradation of phospho-beta-catenin, thereby increasing Wnt signalling pathway essential in the formation of osteoblasts [23].

**Figure 1:**
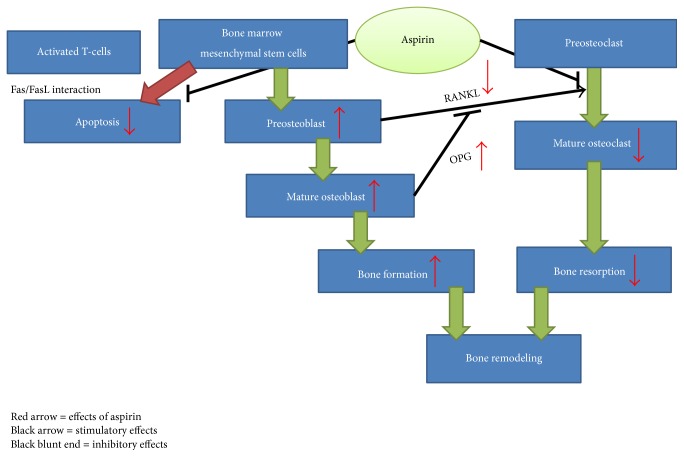
The effects of aspirin on bone cells.

In summary, aspirin could prevent the formation of osteoclast through inhibition of NF*κ*B pathway and enhance the formation of osteoblast by preventing apoptosis of its progenitor stem cell and stimulating the differentiation of preosteoblast (Figure 1).

## 4. The Effects of Aspirin on Animal Model of Osteoporosis

The earliest animal study examining the effects of aspirin on bone was conducted by Waters et al. In their study, 14-week-old female dogs subjected to hind-limb immobilization were treated with 25 mg/kg aspirin every eight hours for 28 days [24]. It was observed that immobilization caused a significant decreased in the normalized bone content of the tibial metaphyseal region and an increase in PGE level in the bone of the immobilized limb compared to the mobile limb [24]. Aspirin treatment reduced the rate of bone loss and PGE level significantly in these animals [24].

Ovariectomy-induced osteoporosis is a popular animal model of bone loss because it is representative of the most common cause of osteoporosis in human, that is, postmenopausal osteoporosis. Chen et al. administered aspirin at the doses of 8.93, 26.79 and 80.36 mg/kg/day to three-month-old ovariectomized rats [25]. All three treatment groups showed significantly higher vertebral bone mineral density (BMD) value compared to ovariectomized control. X-ray microtomography (micro-CT) also revealed significant improvements in bone structural indices and volumetric BMD in rats treated with the three doses of aspirin [25]. The positive changes in bone structure were translated to a higher biomechanical strength of the bone, as indicated by increased vertebral and femoral load value of the treated rats compared to ovariectomized controls [25]. The effects of aspirin on bone as shown in this study were dose-dependent [25].

Yamaza et al. showed that treating mice for three months with 0.6 mg/mL aspirin prevented the degeneration of trabecular and cortical density due to ovariectomy [23]. The mice were ovariectomized one month before sacrifice. Aspirin treatment could reduce serum RANKL and increase OPG level [23]. Immunohistochemical staining showed a marked reduction in the number of cells stained positively with TRAP in the tibia of mice treated with aspirin, indicating less osteoclast formation [23].

The combination effects of aspirin with other antiosteoporotic regimes (hormones and stem cell therapy) have been scrutinized by several research groups. The bone protecting effects between diethylstilboestrol (DES) (30 *μ*g/kg/day) and the combination of DES (10 *μ*g/kg/day) and aspirin (nine mg/kg/day) for 90 days in four-month-old ovariectomized rats were compared [26]. Diethylstilboestrol was synthetic oestrogen used in this study to mimic oestrogen replacement therapy in human. Both treatment groups demonstrated improvements in bone structural indices and a reduction in osteoclast number and perimeter assessed using bone histomorphometric techniques [26]. However, only rats treated with high-dose DES showed a significant increase in percentage of labelled perimeter [26]. Bone mineral density of the left femur was significantly higher in both groups compared with ovariectomized controls [26]. In the same study, lipid profile of rats was measured [26]. Both groups lowered total cholesterol but did not affect high-density lipoprotein cholesterol level [26]. The low-density lipoprotein lowering effect of the combination therapy was greater than high-dose DES [26]. In addition, only the combination therapy reduced triglyceride level in the rats [26].

Wei et al. examined the effects of salmon calcitonin (2 U/kg/day), aspirin (34.4 mg/kg/day), or both for 12 weeks on bone in three-month old ovariectomized rats [27]. All three treatment regimens prevented the decline of vertebral BMD due to oestrogen deficiency, but the effect of combined treatment was superior compared to individual treatments [27]. Increased bone turnover, marked by serum bone formation markers alkaline phosphatase, procollagen type I C-terminal propeptide and osteocalcin, and bone resorption marker type I collagen cross-linked telopeptide, was suppressed in all three treatment groups [27]. Aspirin alone and the combined treatment also augmented femoral stiffness and ultimate load [27]. The bone protective action of aspirin in this study was attributed to decreased stimuli for osteoclast formation, marked by a reduction in mRNA and protein expression for RANKL in the bone [27]. On the other hand, calcitonin was able to increase mRNA and protein expression of OPG in the bone. Rats treated with the combined regimes benefited from both actions, thereby possessing a greater OPG/RANKL ratio compared to individual treatments [27].

Liu at al. treated eight-week-old rats with established bone loss (ovariectomized four weeks before treatment) with allogeneic adipose stem cells (6 × 10^6^ cells; administered four times throughout the treatment period), aspirin (100 mg/kg body weight; daily), or aspirin plus stem cells for 12 weeks [7]. Aspirin alone improved bone structural indices assessed with micro-CT but did not alter bone formation rate and bone turnover markers compared to ovariectomized controls [7]. It also increased serum calcium level and lowered inflammatory cytokine levels (tumour necrosis alpha and interferon gamma) significantly [7]. Rats receiving stem cell per se experienced similar changes as the aspirin group, with greater improvements in bone formation rate and procollagen I N-terminal peptide (bone formation marker) [7]. The positive alternations caused by the combined treatments changes surpassed individual treatments. Further studies showed that aspirin facilitated the migration and homing of stem cells [7]. In a related study, Yamaza et al. indicated that aspirin could increase the bone forming capacity of immunocompromised rats transplanted with bone marrow mesenchymal stem cells [23].

Overall, animal studies in general indicate a bone protective effect of aspirin. Aspirin can be used as an individual treatment or in combination with other bone protective therapies (drugs or stem cells) to prevent bone loss in animals. The use of ovariectomized sexually matured young rats (<6 months old) as a model of bone loss remains controversial because rats reach skeletal maturity later in life (approximately 12 months old). Hence, the model of ovariectomized young rats is similar to a model of stunned skeletal growth rather than degeneration [28]. The dose of aspirin used in the aforementioned studies varies greatly. After converting into human equivalent dose using formula based on body surface area [29], the dose used by Waters was well above the safety margin, while the rest were below the recommended dose used in primary prevention of cardiovascular disease in human (100 mg per day or below) (Table 1) [14]. None of the studies examined the bleeding tendency of the rats, thus safety of the dose could not be confirmed.

## 5. The Relationship between Aspirin Use and Bone Health in Epidemiological Study

In the multicentred Study of Osteoporotic Fractures involving 7,786 Caucasian women aged 65 years and above, Bauer et al. observed that BMD at hip and spine was higher in women using aspirin 5–7 times/week compared to nonusers after adjusting for confounders [30]. Women who used aspirin for more than a year also had higher BMD at hip and spine compared to nonusers [30]. However, the use of aspirin was not associated with four-year fracture risk at the hip [relative risk ratio: 1.1 (95% CI: 0.7–1.6)] or all nonspinal fractures [relative risk ratio: 1.0 (95% CI: 0.8–1.2)] [30]. This study was noteworthy for its large sample size and adjustment for confounders like the presence of osteoarthritis, which was known to increase BMD of the patients. Nevertheless, the use of aspirin or NSAID was self-reported, thus recall bias was possible [30]. The findings could be difficult to be extrapolated to other populations because the subjects were all Caucasian women [30].

Similarly in the Danish Osteoporosis Prevention Study involving 2,016 women aged 45–58 years, Vestergaard et al. showed that whole body, lumbar spine, total hip, femoral neck, and distal forearm BMD did not differ between aspirin users and nonusers at baseline [31]. Unadjusted rate of decline in spinal BMD was lower in the aspirin users, but the significance was lost after multiple adjustments [31]. Ten-year follow-up revealed that aspirin use was not associated with fracture risk [hazard ratio: 0.94 (95% CI: 0.66–1.33)] [31]. This was a large study with a relatively long follow-up period. However, the subjects were all Caucasian women so extrapolation of the findings remained an issue.

In the Health, Aging, and Body Composition Study involving 2,853 subjects (50.5% men, 49.5% women; 43.1% African Americans, 56.9% Caucasians) with a mean age of 73.6 years (range 70–19 years), Carbone et al. found that whole body BMD was significantly higher in subjects using aspirin only and in those using aspirin plus relative COX-2 selective NSAIDs [32]. The subjects using both drugs also had higher total hip BMD [32]. Besides, the subjects using aspirin alone or concurrently with relative COX-1 or COX-2 selective NSAIDs had higher cortical and trabecular BMD assessed with quantitative computed tomography [32]. For subjects using the drugs for at least one year, whole body BMD was higher in relative COX-2 selective NSAIDs aspirin users, and hip BMD was higher in relative COX-1 selective NSAIDs aspirin users [32]. The strength of this study was the composition of the subjects, encompassing both men and women and two distinct ethnic groups. However, a causal relationship could be resolved with a cross-sectional study and fracture risk was not included as the end point.

In a case-control study [124,655 cases aged 43.44 (SD = 27.39) years; 373,962 controls aged 43.44 (SD = 27.39) years], Vestergaard et al. found that risk for any fractures decreased with the use of low-dose aspirin [odds ratio: 0.93 (95% CI: 0.91–0.96)] after adjusting for multiple confounders [33]. In adjusted model, aspirin more than one defined daily dose/day (1 DDD/day) was associated with increased risk for any fractures [odd ratio: 1.17 (95% CI: 1.02–1.34)] [33]. Low-dose aspirin at 0.5 or less DDD/day [odds ratio: 1.10 (95% CI: 1.01–1.20)] and between 0.51 and 1 DDD/day [odds ratio: 1.17 (95% CI: 1.08–1.27)] was also associated with osteoporotic hip fracture [33]. Since it was a case-control study, many confounding factors such as compliance of the drug could not be accessed.

Overall, human epidemiological studies suggested a small positive effect of aspirin on BMD. Its effect on fracture risk, however, ranged from nil to higher risk (Table 2). All of these studies are observational; therefore, the findings are at best hypothesis generating. Taking the possibly small effects size and adverse effects of aspirin into consideration, it will be hard to implement a randomized controlled trial to test the effects of aspirin on BMD and fracture risk. More data from large prospective studies are required to confirm the effects of aspirin on bone health.

## 6. Future Research Perspective

Several research gaps are yet to be bridged in the field. Firstly, there is no direct evidence indicating that the bone protective mechanisms of aspirin previously mentioned are dependent on its COX-inhibitory activities. This could be achieved by the use of genetic modified animals, such as COX-knockout mice. Secondly, both COX-2 specific NSAIDs and aspirin are protective of bone health [34]. The efficacy of these two types of NSAIDs should be compared to ascertain the inhibition of which COX subtypes is most beneficial to skeletal health. Thirdly, PGE_2_ is essential for the transduction of mechanical signals in osteocytes [35]. The effects of aspirin on this process should be determined because aspirin can impair PGE_2_ synthesis. Fourthly, the negative effects of oestrogen deficiency on bone health are partially mediated with low-grade inflammation [36]. Further studies should explore whether aspirin is able to cease bone loss by preventing the low-grade inflammation induced by oestrogen depletion. Fifthly, the potential of aspirin as an adjuvant to the current standard osteoporosis therapy should be explored. For example, the standard bone anabolic agent, parathyroid hormone, increases both bone formation and resorption with the former in excess over the latter [37]. The use of aspirin can lower the bone resorption process and enhance the antiosteoporosis effects of parathyroid hormone.

## 7. Conclusion

Evidence from cellular and animal studies suggests that aspirin possesses bone protective effects. Aspirin is able to promote the survival of osteoblast precursor stem cells and differentiation of osteoblast. It also inhibits the NF*κ*B pathway, reduces the expression of RANKL, and increases OPG, thus suppressing the differentiation of osteoclast. Thus, bone health deterioration is prevented in aspirin-treated animal subjected to bone loss. The skeletal effects of aspirin in human are limited and inconclusive. Aspirin may increase bone mineral density of the users, but this does not translate to fracture prevention. Data from more large-scale prospective epidemiological studies are essential in validating the relationship between the use of aspirin and fracture.

## Figures and Tables

**Table 1:** The effects of aspirin on bone in animal models of bone loss.

Authors	Animal model used	Study design/aspirin dose/treatment period	Estimated human equivalent dose for 60 kg adult per day (mg)	Findings	Notes
Waters et al. (1991) [24]	14-week-old female dogs with their right hind limb immobilized using fiberglass cast	The hind limb-immobilized dogs were treated with 25 mg/kg aspirin every 8 hours for 28 days.	24324.32^a^	↓ rate of bone loss ↓ PGE_2_ level	a = assuming weight of the dog was 10 kg and 3 times treatment of aspirin per day

Chen et al. (2011) [25]	3-month-old ovariectomized rats	Rats were treated three months after ovariectomy with aspirin at the dose of 8.93, 26.79, and 80.36 mg/kg/day for three months.	21.72, 65.16, 195.47^b^	↑ BMD ↑ bone structural indices ↑ biomechanical strength No changes in bone formation markers All changes were dose-dependent	b = assuming weight of the rat was 250 g

Lin et al. (2013) [26]	4-month-old ovariectomized rats	The ovariectomized rats were treated with diethylstilbestrol (hi-DES) (30 *µ*g/kg/day) or aspirin (9 mg/kg/day) plus DES (10 *µ*g/kg/day) for 90 days.	21.89^b^	↑ BMD for both groups ↑ bone structural indices for both groups ↓ osteoclast number and perimeter for both groups ↑ % labelled perimeter in hi-DES group ↓ total cholesterol, low-density lipoprotein cholesterol for both groups ↑ triglyceride for hi-DES group No changes in high density lipoprotein cholesterol for both groups	b = assuming weight of the rat was 250 g

Liu et al. (2015) [7]	8-week-old ovariectomized rats	Rats were ovariectomized and treated 4 weeks later. Effects of zoledronate, allogeneic adipose stem cells (ASC) (6 × 10^6^ cells), stem cell media, aspirin (100 mg/kg/day), or aspirin + stem cells were assessed for 12 weeks. Aspirin administered daily and stem cells administered 4 times.	243.24^b^	↑ bone structural indices and serum calcium in aspirin and ASC groups ↑ bone structural indices, bone formation rate, bone formation markers in aspirin + ASC group ↑ migration and homing of ASC	b = assuming weight of the rat was 250 g

Wei et al. (2015) [27]	3-month-old ovariectomized rats	Ovariectomized rats were treated with aspirin 34.4 mg/kg/day, salmon calcitonin (CAL) 2 U/kg/day or both concomitantly for 12 weeks.	83.68^b^	↑ vertebral BMD for all groups No changes in femoral BMD and serum calcium and phosphate level for all groups ↑ biomechanical strength for aspirin group and CAL + aspirin group ↓ bone turnover for all groups ↓ RANKL expression in aspirin and CAL + aspirin group ↑ OPG expression in CAL and CAL + aspirin group	b = assuming weight of the rat was 250 g

Yamaza et al. (2008) [23]	Ovariectomized mice	Mice were fed with aspirin 0.6 mg/ml for three months.	72.97^c^	↑ trabecular and cortical density ↑ serum OPG ↓ serum RANKL ↓ number of TRAP + cells	c = assuming one treatment per day with gavage volume 25 ml

**Table 2:** The association between aspirin use and bone mineral density and fracture risk.

Authors (years)	Characteristics of the subjects	Study design	Findings	
			Bone mineral density (BMD)	Fracture risk
Bauer et al. 1996 [30]	7,786 Caucasian women aged 65 years above from the multicentred Study of Osteoporotic Fractures.	BMD measured cross-sectionally and fractures were followed up after 4 years.	↑	*↔*
Carbone et al. 2003 [32]	2,853 subjects (49.5% women, 50.5% men) aged 73.6 years from the Health, Aging, and Body Composition Study.	BMD measured by DXA and QCT cross-sectionally.	↑	NA
Vestergaard et al. 2012 [31]	2,016 female participants aged 45–58 years from the Danish Osteoporosis Prevention Study.	BMD and fracture of the participant were traced for 10 years.	*↔*	*↔*
Vestergaard et al. 2012 [33]	Cases = 124,655 subjects aged 43.44 (SD = 27.39) years. Control = 373,962 subjects aged 43.44 (SD = 27.39) years.	Case-control study	NA	↑

## References

[1] Raggatt L. J., Partridge N. C. (2010). Cellular and molecular mechanisms of bone remodeling. *Journal of Biological Chemistry*.

[2] Kearns A. E., Khosla S., Kostenuik P. J. (2008). Receptor activator of nuclear factor *κ*B ligand and osteoprotegerin regulation of bone remodeling in health and disease. *Endocrine Reviews*.

[3] Hadjidakis D. J., Androulakis I. I. (2006). Bone remodeling. *Annals of the New York Academy of Sciences*.

[4] Raisz L. G. (2001). Potential impact of selective cyclooxygenase-2 inhibitors on bone metabolism in health and disease. *The American Journal of Medicine*.

[5] Agas D., Marchetti L., Capitani M., Sabbieti M. G. (2013). The dual face of parathyroid hormone and prostaglandins in the osteoimmune system. *American Journal of Physiology—Endocrinology and Metabolism*.

[6] Blackwell K. A., Raisz L. G., Pilbeam C. C. (2010). Prostaglandins in bone: bad cop, good cop?. *Trends in Endocrinology and Metabolism*.

[7] Liu H., Li W., Liu Y., Zhang X., Zhou Y. (2015). Co-administration of aspirin and allogeneic adipose-derived stromal cells attenuates bone loss in ovariectomized rats through the anti-inflammatory and chemotactic abilities of aspirin. *Stem Cell Research and Therapy*.

[8] Suda K., Udagawa N., Sato N. (2004). Suppression of osteoprotegerin expression by prostaglandin e2 is crucially involved in lipopolysaccharide-induced osteoclast formation. *Journal of Immunology*.

[9] Zhou Y., Boudreau D. M., Freedman A. N. (2014). Trends in the use of aspirin and nonsteroidal anti-inflammatory drugs in the general U.S. population. *Pharmacoepidemiology and Drug Safety*.

[10] Cheng B., Kato Y., Zhao S. (2001). PGE_2_ is essential for gap junction-mediated Intercellular communication between osteocyte-like MLO-Y4 cells in response to mechanical strain. *Endocrinology*.

[11] Fuster V., Sweeny J. M. (2011). Aspirin: a historical and contemporary therapeutic overview. *Circulation*.

[12] Paez Espinosa E. V., Murad J. P., Khasawneh F. T. (2012). Aspirin: pharmacology and clinical applications. *Thrombosis*.

[13] Furst D. E., Ulrich R. W., Prakash S., Katzung B. G., Masters S. B., Trevor A. J. (2012). Nonsteroidal anti-inflammatory drugs, disease-modifying antirheumatic drugs, nonopioid analgesics, & drugs used in gout. *Basic and Clinical Pharmacology*.

[14] Bibbins-Domingo K., Grossman D. C., Curry S. J. (2016). Aspirin use for the primary prevention of cardiovascular disease and colorectal cancer: U.S. preventive services task force recommendation statement. *Annals of Internal Medicine*.

[15] Dehmer S. P., Maciosek M. V., Flottemesch T. J., LaFrance A. B., Whitlock E. P. (2016). Aspirin for the primary prevention of cardiovascular disease and colorectal cancer: a decision analysis for the U.S. preventive services task force. *Annals of Internal Medicine*.

[16] Feng X., McDonald J. M. (2011). Disorders of bone remodeling. *Annual Review of Pathology: Mechanisms of Disease*.

[17] Papaioannou A., Kennedy C. C., Ioannidis G. (2009). The impact of incident fractures on health-related quality of life: 5 years of data from the Canadian multicentre osteoporosis study. *Osteoporosis International*.

[18] Ekegren C. L., Edwards E. R., Page R. (2016). Twelve-month mortality and functional outcomes in hip fracture patients under 65 years of age. *Injury*.

[19] Lee S. H., Chen I. J., Li Y. H., Fan Chiang C. Y., Chang C. H., Hsieh P. H. (2016). Incidence of second hip fractures and associated mortality in taiwan: a nationwide population-based study of 95,484 patients during 2006–2010. *Acta Orthopaedica et Traumatologica Turcica*.

[20] Zeng Y. P., Yang C., Li Y. (2016). Aspirin inhibits osteoclastogenesis by suppressing the activation of NF-*κ*B and MAPKs in RANKL-induced RAW264.7 cells. *Molecular Medicine Reports*.

[21] Lawrence T. (2009). The nuclear factor NF-*κ*B pathway in inflammation. *Cold Spring Harbor Perspectives in Biology*.

[22] Tak P. P., Firestein G. S. (2001). NF-*κ*B: a key role in inflammatory diseases. *The Journal of Clinical Investigation*.

[23] Yamaza T., Miura Y., Bi Y. (2008). Pharmacologic stem cell based intervention as a new approach to osteoporosis treatment in rodents. *PLoS ONE*.

[24] Waters D. J., Caywood D. D., Trachte G. J., Turner R. T., Hodgson S. F. (1991). Immobilization increases bone prostaglandin E: effect of acetylsalicylic acid on disuse osteoporosis studied in dogs. *Acta Orthopaedica*.

[25] Chen Z.-W., Wu Z.-X., Sang H.-X. (2011). Effect of aspirin administration for the treatment of osteoporosis in ovariectomized rat model. *Zhonghua yi xue za zhi*.

[26] Lin S. E., Huang J. P., Wu L. Z., Wu T., Cui L. (2013). Prevention of osteopenia and dyslipidemia in rats after ovariectomy with combined aspirin and low-dose diethylstilbestrol. *Biomedical and Environmental Sciences*.

[27] Wei J., Wang J., Gong Y., Zeng R. (2015). Effectiveness of combined salmon calcitonin and aspirin therapy for osteoporosis in ovariectomized rats. *Molecular Medicine Reports*.

[28] Turner R. T., Maran A., Lotinun S. (2001). Animal models for osteoporosis. *Reviews in Endocrine and Metabolic Disorders*.

[29] Reagan-Shaw S., Nihal M., Ahmad N. (2008). Dose translation from animal to human studies revisited. *FASEB Journal*.

[30] Bauer D. C., Orwoll E. S., Fox K. M. (1996). Aspirin and NSAID use in older women: effect on bone mineral density and fracture risk. *Journal of Bone and Mineral Research*.

[31] Vestergaard P., Hermann P., Jensen J.-E. B., Eiken P., Mosekilde L. (2012). Effects of paracetamol, non-steroidal anti-inflammatory drugs, acetylsalicylic acid, and opioids on bone mineral density and risk of fracture: results of the danish osteoporosis prevention study (dops). *Osteoporosis International*.

[32] Carbone L. D., Tylavsky F. A., Cauley J. A. (2003). Association between bone mineral density and the use of nonsteroidal anti-inflammatory drugs and aspirin: impact of cyclooxygenase selectivity. *Journal of Bone and Mineral Research*.

[33] Vestergaard P., Steinberg T. H., Schwarz P., Jørgensen N. R. (2012). Use of the oral platelet inhibitors dipyridamole and acetylsalicylic acid is associated with increased risk of fracture. *International Journal of Cardiology*.

[34] Konstantinidis I., Papageorgiou S. N., Kyrgidis A., Tzellos T.-G., Kouvelas D. (2013). Effect of non-steroidal anti-inflammatory drugs on bone turnover: an evidence-based review. *Reviews on Recent Clinical Trials*.

[35] Liu C., Zhang X., Wu M., You L. (2015). Mechanical loading up-regulates early remodeling signals from osteocytes subjected to physical damage. *Journal of Biomechanics*.

[36] Riggs B. L., Khosla S., Melton L. J. (2002). Sex steroids and the construction and conservation of the adult skeleton. *Endocrine Reviews*.

[37] Aslan D., Andersen M. D., Gede L. B. (2012). Mechanisms for the bone anabolic effect of parathyroid hormone treatment in humans. *Scandinavian Journal of Clinical and Laboratory Investigation*.

